# Invirmination, Associated with Measles

**Published:** 1874-01-01

**Authors:** F. K. Bailey

**Affiliations:** Knoxville, Tenn.


### INVIRMINATION, ASSOCIATED WITH MEASLES. BUBONOCELE.

CLINICAL NOTES, BY F. K. BAILEY, M.D., KNOXVILLE, TENN.

CASE I.—*April 3d*, 1873.—F. O., female, nearly five years old; nervous temperament, and rather feeble organization; fair skin and hair; light brown eyes. Came from Savannah, Ga., ten days ago, where the parents have resided a few months. Complains of pains in the stomach and bowels, with impaired appetite; abdomen full; constipated, and the tongue coated white. Believing that intestinal worms were a source of at least a part of the trouble, gave small powders, as follows:

℞.—Santonin, grs. iv.
Calomel, grs. ij.
Pulv. Doveri, grs. viij.

F., 4 powders. Sig., one every three hours, followed with castor oil on taking the last.

*April 6th*, 10 A. M.—Was called, and learned that nine large round worms had been voided since Thursday. Found the child much depressed; pulse soft, feeble, and about 120; face purplish; and inclined to sleep. Was told that her mother had, during the morning, given her a dessert spoonful of "vermifuge," which I found to be composed, principally, of wormseed oil. Has voided one worm since daylight. Gave milk-punch, and half grain doses of quinia every hour. 12 M.—Has aroused so as to talk; countenance now natural; tongue clearing off; occasional reddish spots upon the face; no cough; and has no pain.

*April 7th*, 9 A. M.—Eruption of measles upon the face and forehead, but slight upon the body. Bowels moved twice during the night; one worm voided. Is wakeful; slept but little during the past night. To have warm drinks and to be kept from a current of air.

*April 11th*.—Has been free from fever since last report; some cough, which was allayed by taking a simple expectorant of syr. ipecac and sang. canadensis. Bowels open.

*April 12th*—Discharged.

In this case the measles were very light; and were she not affected at the same time with worms, no disturbance would have resulted. As it was, the child nearly succumbed to the reflex condition induced by their presence.

CASE II.—W., aged fifteen; pure African; came to my office, and showed a large swelling in the left groin. There is a warty excrescence upon the penis, posteriorly, and near the frænum, which is reddened and appears inflamed. No other lesion of structure. Touched the wart with strong solution of carbolic acid, and directed him to return.

*April 7th*.—A small, soft chancrous abrasion upon the glans can be seen. This I touched with carbolic acid, and I prescribed tinct. iodine to be applied to the inguinal swelling.

*April 26th*.—This boy called in occasionally for a few days, and the sore upon the glans healed readily. The inguinal swelling subsided slowly, without opening.

Of the specific character of the affection above described, I am not sure.

The boy admitted having connection with a girl, as likewise did two or three others, who had simple gonorrhœa, at the same time. It is not at all uncommon to meet with negro boys, of a more tender age than this fellow, laboring under syphilis and gonorrhœa.

In *ante bellum* days, we read of a social status among the slaves which shocked our sensibilities. If such a condition obtained *then*, which was worse than what we know to be true now, if the admission of patients is relied on, the slave condition was truly deplorable. Emancipation certainly has not proved a reformation.

I would not tempt incredulity on the part of the readers of THE EXAMINER in the relation of what is well known to be true; and the same is true, also, of a certain class of whites in our midst.

In illustration of the latter remark, I will relate the following case:

In March, 1871, a woman brought to me her little boy, three years old, who, as she said, another doctor had told her had "a bad disorder." I found phymois, and upon the left side of the glans, bulging out through the preputianal integuments, a hard swelling. This had been touched with some kind of caustic by my predecessor in the case, which caused inflammation, and the phymotic condition as above stated.

I merely advised some soothing application for the present, and soon lost sight of the case. I learned subsequently that some one had him in charge who treated the affection as syphilis. A glance at the social surroundings of the family would suffice to render a diagnosis certain.

I once met with the case of a white girl, not over nine years old, who had to exhibit as profuse a crop of condylomata upon the genitals, and extending well over the whole perinial region, as was ever seen in one of riper years. There appeared to be both gonorrhœa and syphilis in this case. Her mother was a white woman, but cohabiting with a coal-black negro.

